# RELAx – REstricted versus Liberal positive end-expiratory pressure in patients without ARDS: protocol for a randomized controlled trial

**DOI:** 10.1186/s13063-018-2640-5

**Published:** 2018-05-09

**Authors:** Anna Geke Algera, Luigi Pisani, Dennis C. J. Bergmans, Sylvia den Boer, Corianne A. J. de Borgie, Frank H. Bosch, Karina Bruin, Thomas G. Cherpanath, Rogier M. Determann, Arjen M. Dondorp, Dave A. Dongelmans, Henrik Endeman, Jasper J. Haringman, Janneke Horn, Nicole P. Juffermans, David M. van Meenen, Nardo J. van der Meer, Maruschka P. Merkus, Hazra S. Moeniralam, Ilse Purmer, Pieter Roel Tuinman, Mathilde Slabbekoorn, Peter E. Spronk, Alexander P. J. Vlaar, Marcelo Gama de Abreu, Paolo Pelosi, Ary Serpa Neto, Marcus J. Schultz, Frederique Paulus, for the RELAx Investigators and the PROVE Network Investigators

**Affiliations:** 1 Department of Intensive Care, Academic Medical Center, Amsterdam, The Netherlands; 2 Department of Intensive Care, Maastricht University Medical Center, Maastricht, The Netherlands; 3 Department of Intensive Care, Spaarne Gasthuis, Haarlem and Hoofddorp, The Netherlands; 4 Clinical Research Unit, Academic Medical Center, Amsterdam, The Netherlands; 5 Department of Intensive Care, Rijnstate, Arnhem, The Netherlands; 6 Department of Intensive Care, Westfriesgasthuis, Hoorn, The Netherlands; 7 Department of Intensive Care, Onze Lieve Vrouwe Gasthuis, Amsterdam, The Netherlands; 8 Madihol–Oxford Research Unit (MORU), Madihol University, Bangkok, Thailand; 9 Department of Intensive Care, Isala Clinics, Zwolle, The Netherlands; 10 Laboratory of Experimental Intensive Care and Anesthesiology (L·E·I·C·A), Academic Medical Center, Amsterdam, The Netherlands; 11 Department of Intensive Care, Amphia Hospital, Breda, The Netherlands; 12 Department of Intensive Care, Sint Antonius Hospital, Nieuwegein, The Netherlands; 13 Department of Intensive Care, Haga Hospital, The Hague, The Netherlands; 14 Department of Intensive Care, VU Medical Center, Amsterdam, The Netherlands; 15 REVIVE Research VU Medical Center, VU Medical Center, Amsterdam, The Netherlands; 16 Department of Intensive Care, Haaglanden Medical Center, The Hague, The Netherlands; 17 Department of Intensive Care, Gelre Hospital, Apeldoorn, The Netherlands; 18 Department of Anesthesiology and Intensive Care, University Hospital Carl Gustav Carus, Dresden, Germany; 19 Department of Surgical Sciences and Integrated Diagnostics, San Martino Policlinico Hospital – IRCCS for Oncology, University of Genoa, Genoa, Italy; 20 Department of Intensive Care Medicine, Hospital Israelita Albert Einstein, São Paulo, Brazil

**Keywords:** Artificial ventilation, Invasive ventilation, Mechanical ventilation, Positive end-expiratory pressure, PEEP, Non-injured lungs, Intensive care unit, Critical care, Duration of ventilation, Mortality

## Abstract

**Background:**

Evidence for benefit of high positive end-expiratory pressure (PEEP) is largely lacking for invasively ventilated, critically ill patients with uninjured lungs. We hypothesize that ventilation with low PEEP is noninferior to ventilation with high PEEP with regard to the number of ventilator-free days and being alive at day 28 in this population.

**Methods/Design:**

The “REstricted versus Liberal positive end-expiratory pressure in patients without ARDS” trial (RELAx) is a national, multicenter, randomized controlled, noninferiority trial in adult intensive care unit (ICU) patients with uninjured lungs who are expected not to be extubated within 24 h. RELAx will run in 13 ICUs in the Netherlands to enroll 980 patients under invasive ventilation. In all patients, low tidal volumes are used. Patients assigned to ventilation with low PEEP will receive the lowest possible PEEP between 0 and 5 cm H_2_O, while patients assigned to ventilation with high PEEP will receive PEEP of 8 cm H_2_O. The primary endpoint is the number of ventilator-free days and being alive at day 28, a composite endpoint for liberation from the ventilator and mortality until day 28, with a noninferiority margin for a difference between groups of 0.5 days. Secondary endpoints are length of stay (LOS), mortality, and occurrence of pulmonary complications, including severe hypoxemia, major atelectasis, need for rescue therapies, pneumonia, pneumothorax, and development of acute respiratory distress syndrome (ARDS). Hemodynamic support and sedation needs will be collected and compared.

**Discussion:**

RELAx will be the first sufficiently sized randomized controlled trial in invasively ventilated, critically ill patients with uninjured lungs using a clinically relevant and objective endpoint to determine whether invasive, low-tidal-volume ventilation with low PEEP is noninferior to ventilation with high PEEP.

**Trial registration:**

ClinicalTrials.gov, ID:NCT03167580. Registered on 23 May 2017.

**Electronic supplementary material:**

The online version of this article (10.1186/s13063-018-2640-5) contains supplementary material, which is available to authorized users.

## Background

Invasive ventilation is a potentially harmful intervention in critically ill patients. Ventilation with low tidal volumes prevents harm by avoiding lung overdistension [1]. The benefit of ventilation with low tidal volumes has clearly been demonstrated in randomized controlled trials (RCTs) in patients with acute respiratory distress syndrome (ARDS) [2], and has strongly been suggested in studies of intensive care unit (ICU) patients with uninjured lungs [3, 4]. Ventilation with high positive end-expiratory pressure (PEEP) prevents harm by minimizing repetitive opening and closing of collapsed lung tissue, but could also induce harm as high PEEP may induce overdistension [1]. The benefit of ventilation with high PEEP was demonstrated in a meta-analysis using individual patient data from RCTs in ARDS patients [5]. However, benefit was only found in patients with moderate or severe ARDS, while harm was found in patients who were classified as having mild ARDS. Of note, while we remain inconclusive with regard to benefit or harm of PEEP in ICU patients with uninjured lungs [6], several reports show that high PEEP is increasingly used in ICUs worldwide, and also in patients without ARDS [7, 8].

The balance between benefit and harm of high PEEP, and actually any level of PEEP, could very well depend on presence and severity of lung injury. In patients with moderate or severe ARDS, in whom large parts of the lungs are collapsed, high PEEP may mainly result in lung recruitment. In patients with mild ARDS, in whom lung collapse is usually less extensive, high PEEP may result in recruitment but at the same time it may induce overdistension. In critically ill patients with uninjured lungs, in whom lung collapse is mostly minimal, high PEEP may only result in overdistension. High PEEP could also have extrapulmonary effects, and also here the balance between benefit and harm could depend on presence and severity of lung injury. High PEEP could affect the blood circulation, as a concomitant rise of the intrathoracic pressure negatively affects the loading conditions of the heart [9]. When PEEP results in considerable lung recruitment, as can be expected in patients with moderate or severe ARDS, this strategy will reduce afterload of the right side of the heart. However, when PEEP also, or mainly, results in overdistension, as in patients with mild ARDS or in  patients with uninjured lungs, afterload of the right side of the heart actually increases. This all may explain why the benefit of high PEEP seems to depend on ARDS severity [5], and why high PEEP, compared to low PEEP or no PEEP, seems to have no benefit in ICU patients with uninjured lungs [6].

**Fig. 1:**
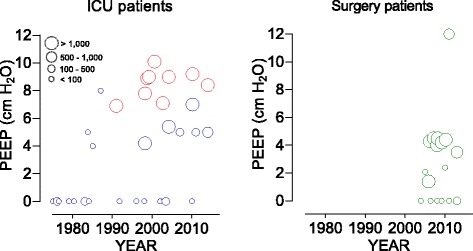
Change in positive end-expiratory pressure (PEEP) in intensive care unit (ICU) and surgical patients over the past years. The practice of PEEP changed remarkably over the last 40 years in ICU patients. As in ICU patients with acute respiratory distress syndrome (ARDS) (red symbols), in ICU patients with uninjured lungs (blue symbols) and in surgery patients (green symbols) high PEEP is increasingly used, despite the lack of evidence. Data are (mean or median) levels of PEEP reported in observational studies, or in the control arms of randomized controlled trials (RCTs) versus the year of start of data collection, or year of publication if the latter was not presented. Abbreviations: *ICU* intensive care unit, *PEEP* positive end-expiratory pressure, *RCTs* randomized controlled trials

Seen the increased use of high PEEP over recent years [6, 7, 10–50] (Fig. 1) and the continued uncertainty as to whether high PEEP truly benefits ICU patients with uninjured lungs, we decided to perform the “REstricted versus Liberal positive end-expiratory pressure in patients without ARDS (RELAx)” trial. In RELAx we test the hypothesis that low-tidal-volume-ventilation with low PEEP is comparably effective as high PEEP in ICU patients with uninjured lungs.

## Methods

### Trial design

RELAx is an investigator-initiated, national, multicenter, open, randomized controlled, noninferiority trial in intubated and ventilated adult ICU patients who do not have ARDS and who are expected to need invasive ventilation for at least 24 h. RELAx will be conducted according to the principles of the Declaration of Helsinki as stated in the current version of Fortaleza, Brazil, 2013 [51], in accordance with the Medical Research Involving Human Subjects Act (WMO) and the International Conference on Harmonization Good Clinical Practice (ICH-GCP) guidelines. The Institutional Review Board of the Academic Medical Center, Amsterdam, the Netherlands, approved the trial protocol (version 3.0, date: 28 July 2017). The trial is registered at www.clinicaltrials.gov (NCT03167580).

**Fig. 2:**
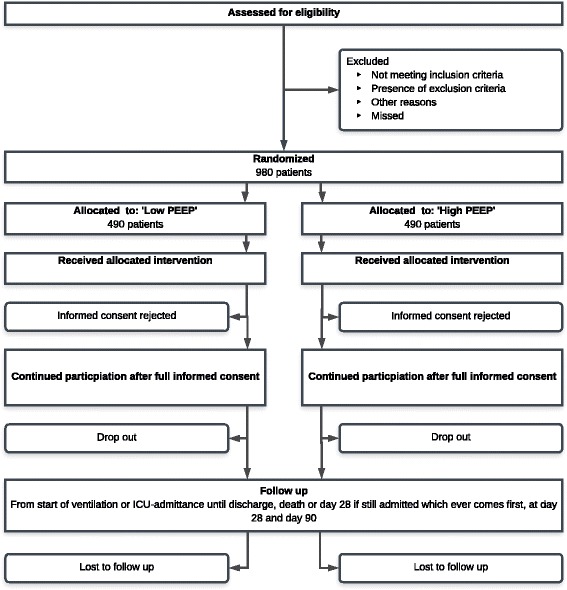
Consolidated Standards of Reporting Trials (CONSORT) diagram. Abbreviation: *PEEP* positive end-expiratory pressure

Patients will be provisionally included under a strategy of deferred consent (see below) and will be randomly assigned to one of the two ventilation strategies described in detail below and presented in Fig. 2. The Standard Protocol Items: Recommendations for Interventional Trials (SPIRIT) 2013 Checklist is provided as Additional file 1.

### Setting

RELAx is performed in the ICU of 13 centers in the Netherlands: three academic centers (Maastricht University Medical Center in Maastricht, VU Medical Center in Amsterdam, Academic Medical Center in Amsterdam) and ten non-academic centers (Amphia Hospital in Breda, Gelre Hospital in Apeldoorn, Haaglanden Medical Center in The Hague, Haga Hospital in The Hague, Isala Clinics in Zwolle, Onze Lieve Vrouwe Gasthuis in Amsterdam, Rijnstate in Arnhem, Sint Antonius Hospital in Nieuwegein, Spaarne Gasthuis in Haarlem and Hoofddorp and Westfriesgasthuis in Hoorn).

### Study population

RELAx will enroll consecutive intubated and ventilated ICU patients without ARDS at onset of ventilation who are expected to need invasive ventilation for longer than 24 h. Patients are screened for eligibility and randomized within 1 h after initiation of invasive ventilation, or within 1 h after admission if invasive ventilation started before entering the ICU. Notably, patients who received invasive ventilation for longer than 12 h directly preceding the present ICU admission are excluded from participation. Patients who have ARDS according to the Berlin definition [52] or severe hypoxemia (PaO_2_/FiO_2_ < 200 mmHg) are also excluded from participation. Other exclusion criteria are age < 18 years, ongoing cardiac ischemia due to cardiac infarction and failed revascularization, increased and uncontrollable intracranial pressure (of ≥ 18 mmHg), delayed cerebral ischemia after subarachnoid hemorrhage, necrotizing fasciitis, and severe untreatable anemia such as in case of Jehovah’s Witnesses. Patients previously randomized in RELAx or participating in another RCT with the similar clinical endpoint or interventions possibly compromising the primary outcome, patients with suspected or confirmed pregnancy, patients with morbid obesity (Body Mass Index > 40 kg/m^2^), patients suffering from GOLD classification III or IV chronic obstructive pulmonary disease (COPD), patients with premorbid restrictive pulmonary disease (evidence of chronic interstitial infiltration on chest radiographs), patients in whom pulse oximetry is known to be unreliable (e.g., patients with carbon monoxide poisoning), and patients with a neurologic diagnosis that can prolong duration of mechanical ventilation (e.g., patients with Guillain-Barré syndrome, high spinal cord lesion or amyotrophic lateral sclerosis, multiple sclerosis, or myasthenia gravis) are also excluded.

### Randomization and blinding

Randomization will be performed using a dedicated, password-protected, SSL-encrypted website. Randomization sequence is generated by a dedicated computer randomization software program (ALEA, TenALEA consortium, Amsterdam, the Netherlands) using random block sizes (maximum size of 8). Due to the nature of the investigational treatment, blinding is not possible. All analyses will be performed in a blinded fashion.

### Deferred consent

For this study, we will include patients using a deferred informed consent since we explicitly want to randomize and accordingly start ventilation within 1 h after start of ventilation, or within 1 h after admission if ventilation was initiated in the emergency or in the operation room. Nevertheless, written informed consent from the legal representative must be obtained as soon as possible thereafter, but never later than 48 h after randomization. If informed consent is not obtained within this time window, or if a legal representative denies participation within this time frame, the patient is excluded and their data will no longer be used.

### The ventilation strategies to be compared

**Fig. 3:**
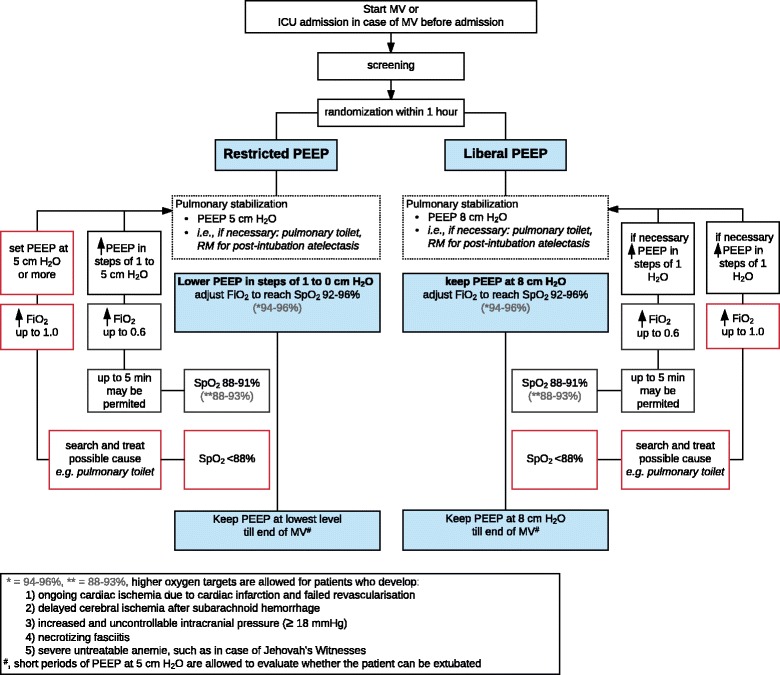
Flowchart ventilator settings with the two ventilation strategies. Abbreviations: *PEEP* positive end-expiratory pressure, *MV* mechanical ventilation, *PBW* predicted body weight, *RM* recruitment maneuver

Patients assigned to receive low PEEP start with PEEP at 5 cm H_2_O and with an inspired oxygen fraction (FiO_2_) between 0.21 and 0.6. Every 15 min PEEP is reduced by 1 cm H_2_O, as long as the pulse oximetry reading shows an oxyhemoglobin saturation by pulse oximetry (SpO_2)_ > 92% or the arterial blood gas shows a PaO_2_ > 8 kPa (Fig. 3). Thereafter, ventilation continues with the lowest PEEP at which the SpO_2_ > 92% or PaO_2_ > 8 kPa, while using a FiO_2_ of between 0.21 and 0.6. These “down-titrations” of PEEP are allowed as often as wanted, but with a minimum of three per ICU nurse shift. When the SpO_2_ drops below 92% or the PaO_2_ drops below 8 kPa, brief periods of 5 min may be tolerated, first FiO_2_ is increased up to maximum 0.6 before PEEP is increased in steps of 1 cm H_2_O until 5 cm H_2_O. As soon as the patient stabilizes, again PEEP is reduced in steps of 1 cm H_2_O to a minimum 0 cm H_2_O. In case of severe hypoxemia, defined as a drop in SpO_2_ below 88% or a drop in PaO_2_ below 7.3 kPa, common causes, such as a mucus plug requiring pulmonary toilet, should be considered and treated. As a pulmonary rescue, the FiO_2_ is increased up to 1.0 and PEEP is set back at 5 cm H_2_O or more (both to a level left to the discretion of the attending physician). After solving the cause for the drop in SpO_2_ or PaO_2_, PEEP is again “down-titrated”, following the same steps as described above. Development of atelectasis, or increases in the amount of atelectasis is not necessarily a reason for increasing PEEP, unless the SpO_2_ drops below 92% or the PaO_2_ drops below 8 kPa, and does not respond to increases in FiO_2_ to maximal 0.6. In case of hemodynamic instability of the patient, meaning that more inotropes and/or vasoactive agents are needed, hemodynamic compromise due to increases in atelectasis could be considered. Then, for a short period of time (e.g., for 1 to 2 h) PEEP can be set at 5 cm H_2_O. After solving the hemodynamic problem, PEEP is again “down-titrated”.

For patients assigned to receive high PEEP, PEEP is set at 8 cm H_2_O with a FiO_2_ between 0.21 and 0.6. The goal is not to change PEEP. In particular, in case the SpO_2_ drops below 92% or the PaO_2_ drops below 8 kPa, first FiO_2_ is increased to maximum 0.6 before PEEP is further increased. In case of severe hypoxemia, defined as a drop in SpO_2_ below 88% or a drop in PaO_2_ below 7.3 kPa, common causes, such as a mucus plug requiring pulmonary toilet, can be considered and treated. As pulmonary rescue, the FiO_2_ is increased up to 1.0 to a level left to the discretion of the attending physician, if necessary PEEP can be increased. After solving the cause for the drop in SpO_2_ or the drop in PaO_2,_ FiO_2_ and PEEP are set back. In case of hemodynamic instability of the patient, meaning that more inotropes and/or vasoactive agents are needed, hemodynamic compromise due to increases in overdistension could be considered. Then, for a short period of time (e.g., for 1 to 2 h) PEEP can be set at 5 cm H_2_O. After solving the hemodynamic problem, PEEP is again set back to 8 cm H_2_O.

### Oxygenation targets

The oxygenation target ranges for SpO_2_ and PaO_2_ are 92 to 96%, and 8 to 11.5 kPa, respectively [53–57]. Oxygenation will be maintained in the target ranges primarily by adjusting the FiO_2_, which is typically set between 0.21 and 0.6. The oxygenation target is primarily assessed by peripheral saturation (SpO_2_) as measured by pulse oximetry and only in case of unreliable readings the oxygenation will be assessed by the arterial blood oxygen pressure (PaO_2_).

For patients in whom the risk of potentially dangerous hypoxemia could be become unacceptable during the trial (e.g., in patients who develop: ongoing cardiac ischemia due to cardiac infarction and failed revascularization, delayed cerebral ischemia after subarachnoid hemorrhage, increased and uncontrollable intracranial pressure (of ≥18 mmHg), necrotizing fasciitis or severe untreatable anemia such as in case with Jehovah’s Witnesses), the target ranges for oxygenation can be increased to SpO_2_ and PaO_2_ of 94 to 96%, and 9 to 11.5 kPa, respectively.

### Standard ventilatory management

The commonly used ventilator modes (volume controlled ventilation, pressure controlled ventilation, and pressure support ventilation) are highly recommended, but all ventilator modes are allowed as long as they do not automatically adjust PEEP and FiO_2_. Tidal volume size is between 6 and 8 ml/kg predicted body weight (PBW), which is calculated according to the following formula [58]: 50 + 0.91 × (centimeters of height − 152.4) for men and 45.5 + 0.91 × (centimeters of height − 152.4) for women. The respiratory rate is adjusted to obtain a normal arterial blood pH (7.35 to 7.45). In case of metabolic acidosis or alkalosis, a lower or higher than normal PaCO_2_ can be accepted, which is left to the discretion of the attending physician. Recruitment maneuvers are allowed when deemed necessary, but the decision to perform a recruitment maneuver is also left to the discretion of the attending physician.

### Ventilator settings when a patient develops ARDS

In case a patient develops ARDS, ventilation should be continued according to existing guidelines for patients with ARDS. This at least consists of low tidal volumes (6 ml/kg PBW or lower) and higher PEEP levels (10 cm H_2_O or higher).

### Weaning from ventilation

Physicians and nurses test every 8 h whether the patient triggers the ventilator, in order to switch to an assisted mode. During assisted ventilation, readiness for extubation will be assessed every 8 h by lowering the pressure support level stepwise to 5 cm H_2_O. Attending physicians decide to extubate a patient based on general extubation criteria, including adequate patient responsiveness and cooperation, appropriate cough reflex, oxygenation saturation > 90% with PaO_2_ to FiO_2_ ratio > 200 mmHg at FiO_2_ ≤ 0.4, and respiratory rate between 8 and 30 breaths per min with no signs of respiratory distress such as marked accessory muscle use, abdominal paradox, diaphoresis or dyspnea. Patients assigned to receive low PEEP are weaned and extubated using the lowest PEEP. Patients assigned to receive high PEEP are weaned and extubated using a PEEP of 8 cm H_2_O. If preferred, PEEP can be set at 5 cm H_2_O for 1 or 2 h directly before extubation, left to discretion of the attending physician.

If a patient is taken *off* mechanical ventilation but subsequently requires additional invasive ventilation within 28 days after randomization, ventilation following the previous assigned PEEP strategy will be restarted.

### Tracheostomy

Tracheostomy is preferably not performed in the first 10 days after intubation. Indications for tracheostomy include expected duration of ventilation > 14 days, Glasgow Coma Score < 7 with inadequate swallow or cough reflex or retention of sputum, severe ICU-acquired weakness evaluated by clinical inspection, and repeated respiratory failure after successive extubations.

### Standard procedures

Sedation follows the local guidelines for sedation in each participating ICU. In general, these guidelines favor the use of analgo-sedation over hypno-sedation, use of bolus over continuous infusion of sedating agents, and the use of sedation scores.

Nurses determine the level of sedation at least three times per day. The adequacy of sedation in each patient is evaluated using a Richmond Agitation Sedation Scale (RASS) [59, 60]. A RASS score between − 2 and 0 is seen as adequate sedation. The goals of sedation are to reduce agitation, stress, and fear; to reduce oxygen consumption (heart rate, blood pressure and minute volume are measured continuously); and to reduce physical resistance to, and fear of, daily care and medical examination. Patient comfort is the primary goal.

To prevent nosocomial infections, selective oropharyngeal decontamination (SOD) or selective decontamination of the digestive tract (SDD) is performed in all patients who are expected to need ventilation for longer than 48 h, and/or are expected to stay in ICU for longer than 72 h [61].

Thrombosis prophylaxis is indicated for all patients who are not treated with anticoagulants; e.g., for therapeutic reasons or systemic prophylaxis because of an implanted device or extra-corporal circulation such as for renal replacement therapy. Thrombosis prophylaxis will be given according to local guidelines.

A fluid balance is targeted at normovolemia and a diuresis of ≥ 0.5 ml/kg/h should be maintained. Crystalloid infusions are preferred over colloid infusions.

A hypocaloric, protein-rich diet (1.2–1.7 g/kg bodyweight per 24 h) is started as soon as possible after ICU admission. Enteral nutrition with a feeding gastric tube is preferred over intravenous feeding. If stomach retention occurs, a duodenal tube can be used if administration of prokinetic drugs is not sufficient, according to local guidelines. When optimal protein intake cannot be reached within 4 days, additional parenteral nutrition can be started.

### Follow-up

**Fig. 4:**
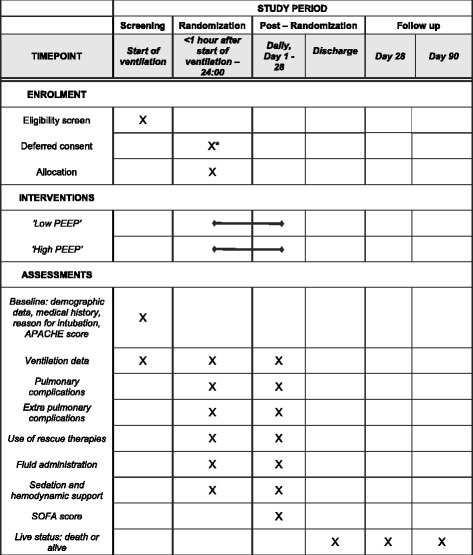
Schedule of enrollment, intervention and assessments. Abbreviations: *APACHE* Acute Physiology And Chronic Health Evaluation, *SOFA* Sepsis-related Organ Failure Assessment score. *Deferred consent, obtained as soon as possible after randomization, but never later than 48 h after randomization

Figure 4 shows the schedule of enrollment, interventions, and assessments. On ICU admission and within the first 24 h, demographic and baseline data, as well as data on disease severity are collected. Data collection includes: gender, age, height, weight, reason for ICU admission, reason for ventilation support, cause of respiratory failure, the Acute Physiology And Chronic Health Evaluation (APACHE) II, IV score and/or the SAPS II.

Data on standard of care and clinical outcome variables (described below) are collected on a daily basis every day until day 28, discharge of the ICU or death, whatever comes first. Data on length of stay (LOS) in the ICU and in the hospital, location of the patient (in ICU, hospital, other facility, or home) and life status (alive or deceased) are assessed on days 28 and 90.

The following variables are collected daily: respiratory status; intubation status (if extubated: time of extubation); tracheostomy status (if tracheostomized: time of tracheostomy, and weaning status: intermittent ventilation via tracheostomy or weaned); development of pulmonary complications (ARDS, severe hypoxemia, pneumonia, severe atelectasis, pneumothorax); need for rescue therapies for severe hypoxemia or severe atelectasis (recruitment maneuver, prone positioning, bronchoscopy for opening atelectasis); days with use of hemodynamic support; days with use of sedation and ICU-acquired weakness [62].

The following mechanical ventilation parameters are collected within 1 h before and 1 h after randomization, and every day at fixed time points until liberation from the ventilator: mode of ventilation, tidal volume, respiratory rate, level of PEEP, FiO_2_, SpO_2_, peak and plateau pressures (volume controlled modes), or maximum airway pressure and level of pressure support above PEEP (pressure controlled modes and pressure support modus) and inspired oxygen fraction.

ICU-related therapy variables to collect daily include: arterial blood gas analysis (once daily), amount and type of infused products including blood products and fluids (crystalloids and colloids), cumulative fluid balance and Sequential Organ Failure Assessment (SOFA) score.

### Study endpoints

The primary endpoint is the number of ventilator-free days and alive at day 28, defined as the number of days from day 1 to day 28 that the patient is alive and breathes without assistance of the mechanical ventilator. A patient must be free of ventilation for at least 24 consecutive hours to have one ventilator-free day; patients who die or are mechanically ventilated more than 28 days are assigned zero ventilator-free days.

Secondary endpoints include ICU- and hospital length of stay (LOS); ICU-, hospital-, and 90-day mortality; incidence of pulmonary complications: development of ARDS, severe hypoxemia, severe atelectasis, pneumothorax, pneumonia; incidence of rescue strategies for severe hypoxemia or severe atelectasis (recruitment maneuver; prone positioning; bronchoscopy for opening atelectasis); days with use of hemodynamic support; days with use of sedation.

### Sample size calculation

The sample size calculation is focused on demonstrating noninferiority. For this calculation, we estimated the duration of invasive ventilation and the associated coefficient of variation to be 5 and 0.7 days, respectively. This was based on data from two large representative patient cohorts that included patients fulfilling the same inclusion and exclusion criteria of the present trial [63, 64].

We calculated that a sample size of 890 patients (445 patients per group) would have 80% statistical power to show noninferiority of a low PEEP strategy compared to a high PEEP strategy, using a one-sided 0.05 significance level and a noninferiority margin of 10% of the duration of invasive ventilation, assuming no difference in the number of ventilator-free days between the two randomization groups.

The choice for a margin of 10% (0,5 days) is motivated by what we consider acceptable from a clinical point. Practically, this margin means that an increase of > 10% in the duration of mechanical ventilation will reduce the number of ventilator-free days and alive at day 28 with > 12 h (calculated over the expected median duration of mechanical ventilation of 5 days) which will be considered inferior [63, 64]. The sample size is increased by 10% to correct for dropouts, meaning that a total of 980 patients will be included.

### Statistical analysis

The primary endpoint is the number of ventilator-free days and alive at day 28 after ICU admission. The null hypothesis entails that ventilation with low PEEP is inferior by a margin of 10% to ventilation with high PEEP. If the lower bound of the one-sided 95% confidence interval of ventilation with low PEEP does not exceed the 10% margin, the null hypothesis of inferiority is rejected. Depending on the distribution we will use a parametric or nonparametric analysis method to evaluate the confidence interval for the difference between the ventilator-free days of both arms.

The statistical analysis will be based on the intention-to-treat principle. In addition, we will perform a per-protocol analysis to check for robustness of results. The per-protocol group analysis only considers those patients who completed the PEEP titration according to the originally allocated treatment study protocol. In this noninferiority trial, we include a superiority, primary-effect analysis. If the noninferiority criterion is satisfied, a secondary analysis of the primary endpoint for superiority will be conducted. Additionally, time to freedom from mechanical ventilation will be expressed with Kaplan-Meier curves. Differences between both PEEP strategies will be analyzed using the log-rank test.

Regarding secondary endpoints, continuous normally distributed variables will be expressed as frequencies and percentages. Differences between groups in continuous normally distributed variables will be expressed by their means and standard deviations or when not normally distributed, as medians and their interquartile ranges. Categorical variables will be expressed as frequencies and percentages. Differences between groups in continuous variables will be analyzed with Student’s *t* test or, if continuous data is not normally distributed, the Mann-Whitney *U* test will be used. Categorical variables will be compared with the chi-squared test or Fisher’s exact test, as appropriate. Time-dependent data will be expressed with Kaplan-Meier curves. Statistical significance is considered to be at a *p* value < 0.05 with a one- or two-sided test, depending on assessment of either noninferiority or superiority. When appropriate, statistical uncertainty will be expressed by 95% confidence levels.

Sub-analyses are planned to investigate the effects of ventilation with low PEEP versus high PEEP in the following pre-specified subgroups on the primary endpoint; patients in obesity subgroups, patients with pulmonary versus non-pulmonary reasons for intubation and mechanical ventilation, and subgroups based on ventilation parameters, including but not restricted to, tidal volume size, respiratory rate, plateau pressure, and level of pressure support.

All statistical analysis will be performed with the R (R Core Team, 2016) software for statistical computing.

### Study organization

The Steering Committee will provide trial oversight and is composed of the principle investigator, the trial coordinating investigators, the local investigators in the participating ICUs and (inter)national experts of ventilation who contribute to the design and revision of the study protocol. The principle investigator and the trial coordinating investigators are responsible for the daily management of the trial. They provide assistance to the participating sites in trial management, record keepings and data management. Furthermore, trial coordinating investigators will provide training regarding study related procedures for the local staff of the participating centers to improve adherence to the protocol. Local investigators in each site will screen the patients who require mechanical ventilation and check if they are eligible for participation, perform randomization, supervise data collection and ensure adherence to the ICH-GCP guidelines during the trial.

### Data management

All data are coded using patient identification numbers (PINs). The key is kept at the trial sites in a secure place. The data are transcribed by the local investigators into Research Electronic Data Capture (REDCap) a central GCP-proof, Internet-based electronic Case Report Form (CRF). Recorded data, provided with a code, will be stored securely for 15 years in archives of the Academic Medical Center, Amsterdam, The Netherlands. Data will be accessible only by the principle investigator and representatives of the Inspectorate for Healthcare of the Netherlands.

### Monitoring and Safety

An independent monitor is appointed to perform study monitoring. During onsite visits, monitoring will be conducted on the following: progress of the study, rights and well-being of the subjects, completeness and accuracy of the recorded data, verifiability of the recorded data from source documents and compliance with GCP-applicable national regulatory guidelines. Every participating center will be visited shortly after inclusion of the first patients to signalize early aberrant patterns and issues; thereafter, every center will be visited at least once a year.

An independent Data Safety and Monitoring Board (DSMB) watches over the ethics of conducting the study in accordance with the Declaration of Helsinki, monitors safety parameters and the overall conduct of the study. The international DSMB is composed of four independent individuals (I. Martin-Loeches MD PhD, P. Severgnini MD, F. van Haren MD PhD, Prof. A. Artigas MD PhD). The DSMB will meet by conference calls. The first meeting will be scheduled soon after the start of the study, subsequent to this meeting the DSMB will meet every 6 months.

As this study compares two treatment strategies that are used in standard care, additional risks are not expected. Furthermore, the study population consists of critically ill patients, with a high incidence of death or life-threatening events due to the severity of their illness (the hospital mortality in ventilated ICU patients is 21% [8]). Therefore, secondary endpoints that incorporate ventilation-specific complications will be reported to the Institutional Review Board and the DSMB in a line-listing format every 6 months, per PEEP strategy, but blinded for treatment groups. Any report and/or advice of the DSMB will be send to the sponsor of the study, the Academic Medical Center, Amsterdam, The Netherlands. Should the sponsor decide not to fully implement advices of the DSMB, the sponsor will send the advice to the reviewing Institutional Review Board, including a note to substantiate why (part of) the advice of the DSMB will not be followed.

### Amendments

All substantial amendments will be notified to the Institutional Review Board and to the competent authority. Non-substantial amendments (typing errors and administrative changes) will not be notified to the accredited Institutional Review Board and the competent authority, but will be recorded and filed by the sponsor.

## Discussion

RELAx is the first randomized controlled trial that is sufficiently powered to investigate whether low-tidal-volume-ventilation with low PEEP is noninferior to ventilation with high PEEP with regard to a clinically relevant endpoint in ICU patients with uninjured lungs. Seeing the uncertainty regarding the best level of PEEP in these patients [65], a well-powered, high-quality trial that focuses on PEEP in this patient cohort is highly needed.

The strengths of RELAx are the large sample size, the multicenter design, and the inclusion of various types of ICU patients. The use of low tidal volumes in both arms allows us to determine the independent effects of the two different PEEP strategies. Other strengths include the use of a strict weaning protocol and the use of local guidelines for sedation and fluid strategies, factors that may all affect duration of invasive ventilation, independent of PEEP. Furthermore, the use of deferred consent allows us to randomize patients as early as possible, meaning that the period of “uncontrolled” ventilation will be minimal.

In the low PEEP group, we opted to use the lowest possible PEEP while maintaining the oxygenation target, assuming that this would avoid or at least minimize lung overdistension, as well as the potential negative effects of PEEP on the systemic circulation [9, 66, 67]. In the high PEEP group, we choose to use a standard level of PEEP, which is the median PEEP used in these patients [68]. Of note, the recently finished NEBULAE trial clearly shows that patients with uninjured lungs are indeed ventilated with 8 cm H_2_O of PEEP [69]. The inclusion and exclusion criteria of the present trial are similar to those used in the NEBULAE trial. Thus, we considered 8 cm H_2_O of PEEP the best level for comparison.

The primary endpoint is the number of ventilator-free days and alive at day 28, a patient-relevant clinical endpoint that is increasingly used in clinical trials of mechanical ventilation [69–72]. This actually is a composite endpoint of duration of ventilation and mortality: the number of ventilator-free days is defined as the number of days a patient is alive and breathes without assistance of a mechanical ventilator during the first 28 days after ICU admission. Patients who died or are ventilated more than 28 days are assigned zero ventilator-free days.

We chose a noninferiority design to compare ventilation with low PEEP to the current standard practice of high PEEP on the primary endpoint. If the noninferiority criterion is satisfied, we will also conduct a superiority analysis for the primary endpoint. Of note, ventilation with low PEEP could be superior to ventilation with high PEEP with regard to the secondary endpoints.

One important limitation of RELAx is that, due the nature of the intervention, blinding is not possible. This is a potential source of bias. However, the weaning process, which directly influences the primary endpoint, stays within the hand of the attending ICU physicians and nurses who have no specific interest in the trial, and all analyses will be performed in a blinded fashion. Secondly, we may run the risk that the two study groups may show insufficient contrast with regard to the level of PEEP used. However, to maximize the difference in PEEP levels between the groups, a minimum of three “down-titrations” of PEEP per nurse shift in the low PEEP arm is emphasized to be performed.

In conclusion, RELAx is an investigator-initiated randomized controlled trial that is adequately powered to test the hypothesis that a ventilation strategy using low PEEP is noninferior compared to one using high PEEP in ICU patients with uninjured lungs with regard to the number of ventilated-free days and alive at day 28.

## Trial status

RELAx started recruiting patients in October 2017.

## Additional file

Additional file 1: Standard Protocol Items: Recommendations for Interventional Trial (SPIRIT) 2013 Checklist: recommended items to address in a clinical trial protocol and related documents. (PDF 177 kb)

## Abbreviations

APACHE: Acute Physiology And Chronic Health Evaluation
ARDS: Acute respiratory distress syndrome
CRF: Case report form
DSMB: Data Safety and Monitoring Board
GCP: Good Clinical Practice
ICH-GCP: International Conference on Harmonization – Good Clinical Practice
ICU: Intensive care unit
LOS: Length of stay
PBW: Predicted body weight
PEEP: Positive end-expiratory pressure
PIN: Patient identification number
RASS: Richmond Agitation Sedation Scale
REDCap: Research Electronic Data Capture
SOFA: Sequential Organ Failure Assessment
SpO_2_: Oxyhemoglobin saturation by pulse oximetry
VFD: Ventilator-free days

## References

[1] Slutsky AS, Ranieri VM (2014). Ventilator-induced lung injury. N Engl J Med.

[2] Putensen C, Theuerkauf N, Zinserling J, Wrigge H, Pelosi P (2009). Meta-analysis: ventilation strategies and outcomes of the acute respiratory distress syndrome and acute lung injury. Ann Intern Med.

[3] Neto AS, Simonis FD, Barbas CSV, Biehl M, Determann RM, Elmer J (2015). Lung-protective ventilation with low tidal volumes and the occurrence of pulmonary complications in patients without acute respiratory distress syndrome: a systematic review and individual patient data analysis. Crit Care Med.

[4] Serpa Neto A, Simonis FD, Barbas CSV, Biehl M, Determann RM, Elmer J (2014). Association between tidal volume size, duration of ventilation, and sedation needs in patients without acute respiratory distress syndrome: an individual patient data meta-analysis. Intensive Care Med.

[5] Briel M, Meade M, Mercat A, Brower RG, Talmor D, Walter SD (2010). Higher vs lower positive end-expiratory pressure in patients with acute lung injury and acute respiratory distress syndrome: systematic review and meta-analysis. JAMA.

[6] Serpa Neto A, Filho RR, Cherpanath T, Determann R, Dongelmans DA, Paulus F (2016). Associations between positive end-expiratory pressure and outcome of patients without ARDS at onset of ventilation: a systematic review and meta-analysis of randomized controlled trials. Ann Intensive Care.

[7] Bellani G, Laffey JG, Pham T, Fan E, Brochard L, Esteban A (2016). Epidemiology, patterns of care, and mortality for patients with acute respiratory distress syndrome in intensive care units in 50 countries. JAMA.

[8] Neto AS, Barbas CSV, Simonis FD, Artigas-Raventós A, Canet J, Determann RM (2016). Epidemiological characteristics, practice of ventilation, and clinical outcome in patients at risk of acute respiratory distress syndrome in intensive care units from 16 countries (PRoVENT): an international, multicentre, prospective study. Lancet Respir Med.

[9] Luecke T, Pelosi P (2005). Clinical review: positive end-expiratory pressure and cardiac output. Crit Care.

[10] Esteban A, Anzueto A, Frutos F, Alía I, Brochard L, Stewart TE (2002). Characteristics and outcomes in adult patients receiving mechanical ventilation: a 28-day international study. JAMA.

[11] Esteban A, Ferguson ND, Meade MO, Frutos-Vivar F, Apezteguía C, Brochard L (2008). Evolution of mechanical ventilation in response to clinical research. Am J Respir Crit Care Med.

[12] Esteban A, Frutos-Vivar F, Muriel A, Ferguson ND, Peñuelas O, Abraira V (2013). Evolution of mortality over time in patients receiving mechanical ventilation. Am J Respir Crit Care Med.

[13] Amato MB, Barbas CS, Medeiros DM, Magaldi RB, Schettino GP, Lorenzi-Filho G, et al. Effect of a protective-ventilation strategy on mortality in the acute respiratory distress syndrome. N Engl J Med. 1998;338:347–54. Massachusetts Medical Society. 10.1056/NEJM199802053380602 9449727

[14] Brower RG, Lanken PN, MacIntyre N, Matthay MA, Morris A, Ancukiewicz M (2004). Higher versus lower positive end-expiratory pressures in patients with the acute respiratory distress syndrome. N Engl J Med.

[15] Villar J, Kacmarek RM, Pérez-Méndez L, Aguirre-Jaime A (2006). A high positive end-expiratory pressure, low tidal volume ventilatory strategy improves outcome in persistent acute respiratory distress syndrome: a randomized, controlled trial. Crit Care Med.

[16] Meade MO, Cook DJ, Guyatt GH, Slutsky AS, Arabi YM, Cooper DJ (2008). Ventilation strategy using low tidal volumes, recruitment maneuvers, and high positive end-expiratory pressure for acute lung injury and acute respiratory distress syndrome: a randomized controlled trial. JAMA.

[17] Mercat A, Richard J-CM, Vielle B, Jaber S, Osman D, Diehl J-L (2008). Positive end-expiratory pressure setting in adults with acute lung injury and acute respiratory distress syndrome: a randomized controlled trial. JAMA.

[18] Wolthuis EK, Choi G, Dessing MC, Bresser P, Lutter R, Dzoljic M (2008). Mechanical ventilation with lower tidal volumes and positive end-expiratory pressure prevents pulmonary inflammation in patients without preexisting lung injury. Anesthesiology.

[19] Wrigge H, Uhlig U, Zinserling J, Behrends-Callsen E, Ottersbach G, Fischer M (2004). The effects of different ventilatory settings on pulmonary and systemic inflammatory responses during major surgery. Anesth Analg.

[20] Zupancich E, Paparella D, Turani F, Munch C, Rossi A, Massaccesi S (2005). Mechanical ventilation affects inflammatory mediators in patients undergoing cardiopulmonary bypass for cardiac surgery: a randomized clinical trial. J Thorac Cardiovasc Surg.

[21] Fernández-Pérez ER, Sprung J, Afessa B, Warner DO, Vachon CM, Schroeder DR (2009). Intraoperative ventilator settings and acute lung injury after elective surgery: a nested case control study. Thorax.

[22] Hess DR, Kondili D, Burns E, Bittner EA, Schmidt UH (2013). A 5-year observational study of lung-protective ventilation in the operating room: a single-center experience. J Crit Care.

[23] Severgnini P, Selmo G, Lanza C, Chiesa A, Frigerio A, Bacuzzi A (2013). Protective mechanical ventilation during general anesthesia for open abdominal surgery improves postoperative pulmonary function. Anesthesiology.

[24] Lin W-Q, Lu X-Y, Cao L-H, Wen L-L, Bai X-H, Zhong Z-J (2008). Effects of the lung protective ventilatory strategy on proinflammatory cytokine release during one-lung ventilation. Ai Zheng.

[25] Levin MA, McCormick PJ, Lin HM, Hosseinian L, Fischer GW (2014). Low intraoperative tidal volume ventilation with minimal PEEP is associated with increased mortality. Br J Anaesth.

[26] Memtsoudis SG, Bombardieri AM, Ma Y, Girardi FP (2012). The effect of low versus high tidal volume ventilation on inflammatory markers in healthy individuals undergoing posterior spine fusion in the prone position: a randomized controlled trial. J Clin Anesth.

[27] Weingarten TN, Whalen FX, Warner DO, Gajic O, Schears GJ, Snyder MR (2010). Comparison of two ventilatory strategies in elderly patients undergoing major abdominal surgery. Br J Anaesth.

[28] Futier E, Constantin J-M, Paugam-Burtz C, Pascal J, Eurin M, Neuschwander A (2013). A trial of intraoperative low-tidal-volume ventilation in abdominal surgery. N Engl J Med.

[29] Maslow AD, Stafford TS, Davignon KR, Ng T (2013). A randomized comparison of different ventilator strategies during thoracotomy for pulmonary resection. J Thorac Cardiovasc Surg.

[30] Hemmes SNT, de Abreu MG, Pelosi P, Schultz MJ (2013). ESA Clinical Trials Network 2012. Eur J Anaesthesiol.

[31] Feeley TW, Saumarez R, Klick JM, McNabb TG, Skillman JJ (1975). Positive end-expiratory pressure in weaning patients from controlled ventilation. A prospective randomised trial. Lancet.

[32] Schmidt GB, O'Neill WW, Kotb K, Hwang KK, Bennett EJ, Bombeck CT (1976). Continuous positive airway pressure in the prophylaxis of the adult respiratory distress syndrome. Surg Gynecol Obstet.

[33] Weigelt JA (1979). Early positive end-expiratory pressure in the adult respiratory distress syndrome. Arch Surg.

[34] Good JT, Wolz JF, Anderson JT, Dreisin RB, Petty TL (1979). The routine use of positive end-expiratory pressure after open heart surgery. Chest.

[35] Zurick AM, Urzua J, Ghattas M, Cosgrove DM, Estafanous FG, Greenstreet R (1982). Failure of positive end-expiratory pressure to decrease postoperative bleeding after cardiac surgery. Ann Thorac Surg.

[36] Murphy DA, Finlayson DC, Craver JM, Jones EL, Kopel M, Tobia V (1983). Effect of positive end-expiratory pressure on excessive mediastinal bleeding after cardiac operations. A controlled study. J Thorac Cardiovasc Surg.

[37] Marvel SL, Elliott CG, Tocino I, Greenway LW, Metcalf SM, Chapman RH (1986). Positive end-expiratory pressure following coronary artery bypass grafting. Chest.

[38] Pepe PE, Hudson LD, Carrico CJ (1984). Early application of positive end-expiratory pressure in patients at risk for the adult respiratory-distress syndrome. N Engl J Med.

[39] Carroll GC, Tuman KJ, Braverman B, Logas WG, Wool N, Goldin M (1988). Minimal positive end-expiratory pressure (PEEP) may be “best PEEP.”. Chest.

[40] Nelson LD, Civetta JM, Hudson-Civetta J (1987). Titrating positive end-expiratory pressure therapy in patients with early, moderate arterial hypoxemia. Crit Care Med.

[41] Cujec B, Polasek P, Mayers I, Johnson D (1993). Positive end-expiratory pressure increases the right-to-left shunt in mechanically ventilated patients with patent foramen ovale. Ann Intern Med.

[42] Vigil AR, Clevenger FW (1996). The effects of positive end-expiratory pressure of intrapulmonary shunt and ventilatory deadspace in nonhypoxic trauma patients. J Trauma.

[43] Michalopoulos A, Anthi A, Rellos K, Geroulanos S (1998). Effects of positive end-expiratory pressure (PEEP) in cardiac surgery patients. Respir Med.

[44] Dyhr T, Laursen N, Larsson A (2002). Effects of lung recruitment maneuver and positive end-expiratory pressure on lung volume, respiratory mechanics and alveolar gas mixing in patients ventilated after cardiac surgery. Acta Anaesthesiol Scand.

[45] Manzano F, Fernández-Mondéjar E, Colmenero M, Poyatos ME, Rivera R, Machado J (2008). Positive-end expiratory pressure reduces incidence of ventilator-associated pneumonia in nonhypoxemic patients. Crit Care Med.

[46] Holland A, Thuemer O, Schelenz C, van Hout N, Sakka SG (2007). Positive end-expiratory pressure does not affect indocyanine green plasma disappearance rate or gastric mucosal perfusion after cardiac surgery. Eur J Anaesthesiol.

[47] Celebi S, Köner O, Menda F, Korkut K, Suzer K, Cakar N (2007). The pulmonary and hemodynamic effects of two different recruitment maneuvers after cardiac surgery. Anesth Analg.

[48] Lesur O, Remillard M-A, St-Pierre C, Falardeau S (2010). Prophylactic positive end-expiratory pressure and postintubation hemodynamics: an interventional, randomized study. Can Respir J.

[49] Borges DL, Nina VJDS, de AG CM, TEP B, NPD S, Lima IM (2013). Effects of different PEEP levels on respiratory mechanics and oxygenation after coronary artery bypass grafting. Rev Bras Cir Cardiovasc.

[50] Lago Borges D, José da Silva Nina V, Pereira Baldez TE, de Albuquerque Gonçalves Costa M, Pereira dos Santos N, Mendes Lima I (2014). Effects of positive end-expiratory pressure on mechanical ventilation duration after coronary artery bypass grafting: a randomized clinical trial. Ann Thorac Cardiovasc Surg.

[51] Declaration of Helsinki (2014). Ethical Principles for Medical Research Involving Human Subjects.

[52] ARDS Definition Task Force, Ranieri VM, Rubenfeld GD, Thompson BT, Ferguson ND, Caldwell E, et al. Acute respiratory distress syndrome: the Berlin Definition. JAMA. 2012;307(23):2526–2533. 10.1001/jama.2012.5669 22797452

[53] Suzuki S, Eastwood GM, Glassford NJ, Peck L, Young H, Garcia-Alvarez M (2014). Conservative oxygen therapy in mechanically ventilated patients: a pilot before-and-after trial. Crit Care Med.

[54] Panwar R, Hardie M, Bellomo R, Barrot L, Eastwood GM, Young PJ (2016). Conservative versus liberal oxygenation targets for mechanically ventilated patients. A pilot multicenter randomized controlled trial. Am J Respir Crit Care Med.

[55] Helmerhorst HJF, Schultz MJ, van der Voort PHJ, Bosman RJ, Juffermans NP, de Wilde RBP (2016). Effectiveness and clinical outcomes of a two-step implementation of conservative oxygenation targets in critically ill patients: a before and after trial. Crit Care Med.

[56] Girardis M, Busani S, Damiani E, Donati A, Rinaldi L, Marudi A (2016). Effect of conservative vs conventional oxygen therapy on mortality among patients in an intensive care unit: The Oxygen-ICU randomized clinical trial. JAMA.

[57] de Jonge E, Peelen L, Keijzers PJ, Joore H, de Lange D, van der Voort PHJ (2008). Association between administered oxygen, arterial partial oxygen pressure and mortality in mechanically ventilated intensive care unit patients. Crit Care.

[58] Network TARDS (2000). Ventilation with lower tidal volumes as compared with traditional tidal volumes for acute lung injury and the acute respiratory distress syndrome. The Acute Respiratory Distress Syndrome Network. N Engl J Med.

[59] Ely EW, Truman B, Shintani A, Thomason JWW, Wheeler AP, Gordon S (2003). Monitoring sedation status over time in ICU patients: reliability and validity of the Richmond Agitation-Sedation Scale (RASS). JAMA.

[60] Sessler CN, Gosnell MS, Grap MJ, Brophy GM, O'Neal PV, Keane KA (2002). The Richmond Agitation-Sedation Scale: validity and reliability in adult intensive care unit patients. Am J Respir Crit Care Med.

[61] Stoutenbeek CP, van Saene HKF, Little RA, Whitehead A, Working Group on Selective Decontamination of the Digestive Tract (2007). The effect of selective decontamination of the digestive tract on mortality in multiple trauma patients: a multicenter randomized controlled trial. Intensive Care Med.

[62] Stevens RD, Marshall SA, Cornblath DR, Hoke A, Needham DM, de Jonghe B (2009). A framework for diagnosing and classifying intensive care unit-acquired weakness. Crit Care Med.

[63] van Vught LA, Klein Klouwenberg PMC, Spitoni C, Scicluna BP, Wiewel MA, Horn J (2016). Incidence, risk factors, and attributable mortality of secondary infections in the intensive care unit after admission for sepsis. JAMA.

[64] Dutch National Intensive Care Evaluation (NICE) Foundation website. https://www.stichting-nice.nl/. Accessed 16 Mar 2015.

[65] Schmidt MFS, Amaral ACKB, Fan E, Rubenfeld GD (2018). Driving pressure and hospital mortality in patients without ARDS: a cohort study. Chest.

[66] Pinsky MR (1997). The hemodynamic consequences of mechanical ventilation: an evolving story. Intensive Care Med.

[67] Retamal J, Bugedo G, Larsson A, Bruhn A (2015). High PEEP levels are associated with overdistension and tidal recruitment/derecruitment in ARDS patients. Acta Anaesthesiol Scand.

[68] MCO v IJ, Koopmans M, Strauch U, Heines S, Boer den S, Kors BM (2014). Ventilator setting in ICUs: comparing a Dutch with a European cohort. Neth J Med.

[69] van Meenen DMP, van der Hoeven SM, Binnekade JM, et al. Effect of On-Demand vs Routine Nebulization of Acetylcysteine With Salbutamol on Ventilator-Free Days in Intensive Care Unit Patients Receiving Invasive Ventilation: A Randomized Clinical Trial. JAMA. 2018;319(10):993–1001. 10.1001/jama.2018.0949 PMC5885882 29486489

[70] Simonis FD, Binnekade JM, Braber A, Gelissen HP, Heidt J, Horn J (2015). PReVENT—protective ventilation in patients without ARDS at start of ventilation: study protocol for a randomized controlled trial. Trials.

[71] Villar J, Belda J, Blanco J, Suarez-Sipmann F, Añón JM, Pérez-Méndez L (2016). Neurally adjusted ventilatory assist in patients with acute respiratory failure: study protocol for a randomized controlled trial. Trials.

[72] Writing Group for the Alveolar Recruitment for Acute Respiratory Distress Syndrome Trial (ART) Investigators. Effect of Lung Recruitment and Titrated Positive End-Expiratory Pressure (PEEP) vs Low PEEP on Mortality in Patients With Acute Respiratory Distress SyndromeA Randomized Clinical Trial. JAMA. 2017;318(14):1335–1345 10.1001/jama.2017.14171 PMC5710484 28973363

